# Block of both TGF-*β* and IL-2 signaling impedes Neurophilin-1^+^ regulatory T cell and follicular regulatory T cell development

**DOI:** 10.1038/cddis.2016.348

**Published:** 2016-10-27

**Authors:** Liang Li, Shu-Han Yang, Yuan Yao, Yu-Qing Xie, Yan-Qing Yang, Yin-Hu Wang, Xue-Ying Yin, Hong-Di Ma, MEric Gershwin, Zhe-Xiong Lian

**Affiliations:** 1 Liver Immunology Laboratory, Institute of Immunology and School of Life Sciences, University of Science and Technology of China, Hefei 230027, China; 2 Division of Rheumatology, Allergy and Clinical Immunology, University of California at Davis School of Medicine, Davis, CA 95616, USA; 3 Innovation Center for Cell Signaling Network, Hefei National Laboratory for Physical Sciences at Microscale, Hefei 230027, China

## Abstract

Understanding the mechanisms that lead to autoimmunity is critical for defining potential therapeutic pathways. In this regard there have been considerable efforts in investigating the interacting roles of TGF-*β* and IL-2 on the function regulatory T cells. We have taken advantage of dnTGF-*β*RII *Il2ra*^−/−^ (abbreviated as *Il2ra*^−/−^Tg) mouse model, which allows a direct mechanistic approach to define the relative roles of TGF-*β* and IL-2 on Treg development. *Il2ra*^−/−^Tg mice spontaneously developed multi-organ autoimmune diseases with expansion of pathogenic T cells and enhanced germinal center response at 3–4 weeks of age. Importantly, peripheral Treg cells from *Il2ra*^−/−^Tg mice demonstrated an activated Th1-like stable phenotype and normal *in vitro* suppressive function, while thymus Treg increased but manifested decreased suppressive function. Interestingly, neither thymus nor peripheral Treg cells of *Il2ra*^−/−^Tg mice contained Neuropilin-1^+^ or PD-1^hi^ phenotype, resulting in defective follicular regulatory T (Tfr) cell development. Such defective Tfr development led to elevated follicular T helper cells, enhanced germinal center responses and increased plasma cell infiltration. These data demonstrate an important synergetic role of TGF-*β* and IL-2 in the generation, activation and stability of Treg cells, as well as their subsequent development into Tfr cells.

Defining the signaling events that lead to loss of tolerance is critical for understanding autoimmunity. Loss of regulatory T (Treg) cells caused by deficiency of Foxp3, which is a master transcription factor that controls Treg cell development and function, results in fatal autoimmune diseases in both mouse and human.^1, 2, 3^ Thus the precise regulation of Treg cells, which play a critical role in controlling excessive host response and preventing autoimmunity,^4^ needs to be fully defined.

Cytokines can be critical for Treg cell development and function. TGF-*β* is important in inducing Treg cells from conventional CD4^+^ T cells as well as Treg development in thymus, while IL-2 maintains TGF-*β* induced Treg cell stability *in vivo.*^5, 6, 7^ TGF-*β* signaling deficiency results in Akt hyperactivation of thymus Treg cells, which converts Treg cells to a Th1 phenotype and decreased their stability intrinsically.^8^ On the other hand, IL-2 can induce the phosphorylation of STAT5, which is important in induction and maintenance of Foxp3 expression and expanding Treg cells.^9^ dnTGF-*β*RII mice, which express a dominant-negative form of the TGF-*β* receptor type II under the control of the murine CD4 promoter, develop inflammatory infiltration due to T cell hyperactivation.^10^ Treg cells from dnTGF-*β*RII mice are less capable of suppressing T cell activation *in vitro*.^11^ *Il2ra*^−/−^ mice develop autoimmune disorders due to dysregulated Treg cells and polyclonal T and B-cell expansion.^12, 13^ Although plenty of work have revealed the role of cytokines TGF-*β* and IL-2 on Treg cells, how they combine to contribute to Treg development in the thymus and maintenance in the periphery needs to be clearly clarified.

In this study, we investigated the synergetic role of TGF-*β* and IL-2 on Treg cells by crossing dnTGF-*β*RII mice with *Il2ra*^−/−^ mice to generate *Il2ra*^−/−^Tg mice, in which TGF-*β* and IL-2 signaling were both blocked in T cells. *Il2ra*^−/−^Tg mice but not control mice, developed multi-organ autoimmune diseases at 3–4 weeks after birth due to dysregulation of Treg cells. TGF-*β* and IL-2 synergistically influenced the development, activation and stability of both thymus and peripheral Treg cells. Importantly, we found that blocking both TGF-*β* and IL-2 signaling impeded Nrp-1^+^ regulatory T cell and follicular regulatory T cell development, which led to enhanced germinal center responses.

## Results

### *Il2ra*^−/−^Tg mice develop lymphadenopathy and multi-organ inflammation

**Figure 1:**
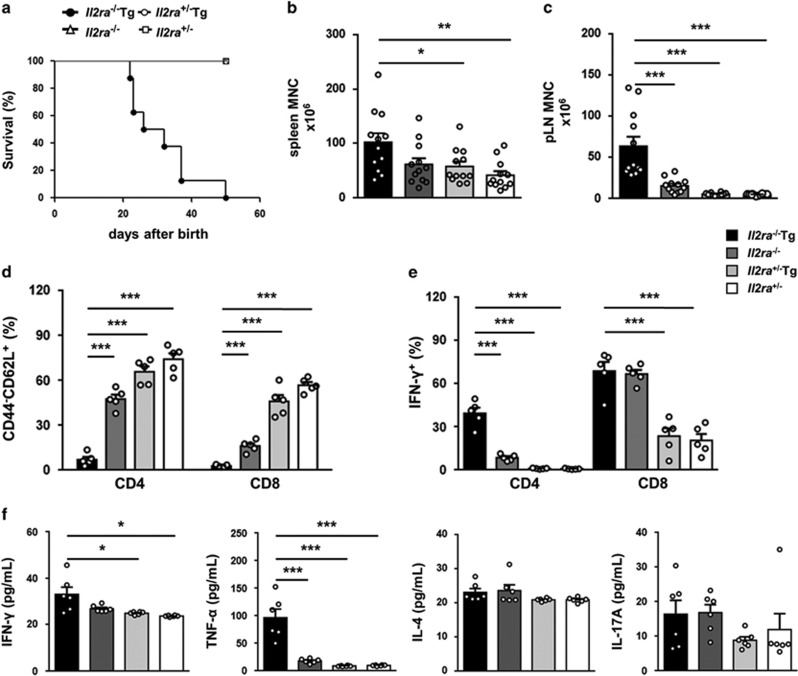
Multi-organ inflammation in *Il2ra*^−/−^Tg mice. (**a**) Survival curve of *Il2ra*^−/−^Tg (*n*=8), *Il2ra*^−/−^ (*n*=8), *Il2ra*^+/−^Tg (*n*=8) and *Il2ra*^+/−^ (*n*=8) mice. Cell counts of (**b**) spleen, (**c**) peripheral (axillary, inguinal and brachial) lymph nodes of *Il2ra*^−/−^Tg (*n*=12), *Il2ra*^−/−^ (*n*=12), *Il2ra*^+/−^Tg (*n*=12) and *Il2ra*^+/−^ (*n*=12) mice. (**d**) Naive T cell (CD44^−^CD62L^+^) percentage and (**e**) IFN-*γ* secreting ability of CD4^+^ and CD8^+^ T cells in pLN from *Il2ra*^−/−^Tg (*n*=5), *Il2ra*^−/−^ (*n*=5), *Il2ra*^+/−^Tg (*n*=5) and *Il2ra*^+/−^ (*n*=5) mice. (**f**) Serum concentration of IFN-*γ*, TNF-*α*, IL-4 and IL-17A of *Il2ra*^−/−^Tg (*n*=6), *Il2ra*^−/−^ (*n*=6), *Il2ra*^+/−^Tg (*n*=6) and *Il2ra*^+/−^ (*n*=6) mice. Data are shown in mean±S.E.M. *P<0.05, **P<0.01, ***P<0.001. (Student's *t* test)

*Il2ra*^−/−^Tg mice began to die at 3 weeks of age while no control mice died during observation (Figure 1a). They were scurfy and had ear atrophy (Supplementary Figure 1a). Increased mononuclear cell number of peripheral lymph nodes (pLNs) and spleen in *Il2ra*^−/−^Tg mice were consistent with lymphadenopathy and splenomegaly (Figures 1b and c and Supplementary Figures 1b and c). Besides, H&E staining revealed intense mononuclear cell infiltration and tissue destruction in liver, pancreas, lung and skin, suggesting multi-organ inflammation. No obvious tissue injury was observed in control mice and *Il2ra*^−/−^ mice had only mild cell infiltration (Supplementary Figure 1d). CD4^+^ and CD8^+^ T cells from *Il2ra*^−/−^Tg mice were more pathogenic, as indicated by decreased naive cell percentage (Figure 1d) as well as increased IFN-*γ* secreting ability (Figure 1e). Moreover, multi-organ inflammation mediated by T cells in *Il2ra*^−/−^Tg mice was accompanied by increased serum levels of TNF-*α* and IFN-*γ*, while IL-4 and the Th17 cytokine IL-17 were similar to littermate controls (Figure 1f).

### Increased follicular helper T cells and enhanced germinal center response in *Il2ra*^−/−^Tg mice

**Figure 2:**
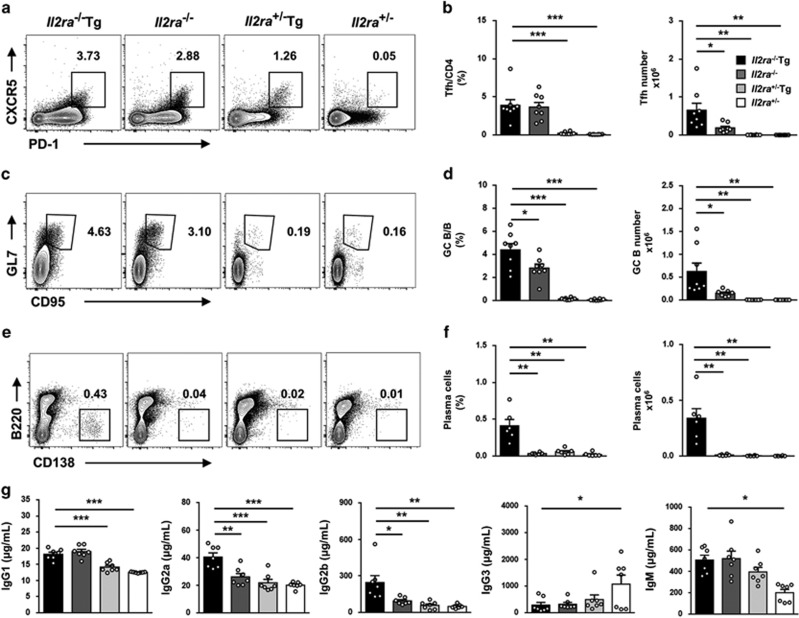
Enhanced germinal center response in *Il2ra*^−/−^Tg mice. Representative flow cytometry result of (**a**) follicular helper T cells (PD-1^hi^CXCR5^+^ in CD4^+^CD19^−^) and (**c**) germinal center B cells (GL7^+^CD95^+^ in CD19^+^) and (**e**) plasma cells (CD19^lo^B220^-^CD138^hi^ in total cells) in pLN of *Il2ra*^−/−^Tg and littermate control mice. (**b**) Follicular helper T-cell percentage in CD4^+^ T cells and absolute number in pLN of *Il2ra*^−/−^Tg (*n*=8), *Il2ra*^−/−^ (*n*=8), *Il2ra*^+/−^Tg (*n*=8) and *Il2ra*^+/−^ (*n*=8) mice. (**d**) Germinal center B-cell percentage in B cells and number in pLN of *Il2ra*^−/−^Tg (*n*=8), *Il2ra*^−/−^ (*n*=7), *Il2ra*^+/−^Tg (*n*=7) and *Il2ra*^+/−^ (*n*=7) mice. (**f**) Plasma cell percentage and number in pLN of *Il2ra*^−/−^Tg (*n*=6), *Il2ra*^−/−^ (*n*=6), *Il2ra*^+/−^Tg (*n*=6) and *Il2ra*^+/−^ (*n*=6) mice. (**g**) Serum concentration of IgG1, IgG2a, IgG2b, IgG3 and IgM of *Il2ra*^−/−^Tg (*n*=7), *Il2ra*^−/−^ (*n*=7), *Il2ra*^+/−^Tg (*n*=7) and *Il2ra*^+/−^ (*n*=7) mice. Data are shown in mean±S.E.M. *P<0.05, **P<0.01, ***P<0.001. (Student's *t* test)

As we observed lymphadenopathy and splenomegaly in *Il2ra*^−/−^Tg mice, we thought to measure the follicular helper T-cell percentage and germinal center response in this mice. We found increased percentage and cell number of Tfh cells in pLN compared with controls (Figures 2a and b). Increased Tfh cells led to enhanced germinal center reactions, as indicated by increased GC B-cell percentage and number in pLN (Figures 2c and d). As a result, plasma cell percentage and number were significantly increased in pLNs of *Il2ra*^−/−^Tg mice compared with littermate controls (Figures 2e and f). Furthermore, there was an increase of IgG1, IgG2a, IgG2b and IgM but decreased IgG3 in *Il2ra*^−/−^Tg serum compared with littermate controls (Figure 2g). These data suggested that antibody secreting plasma cells may play a pathogenic role in multi-organ inflammation and tissue destruction in *Il2ra*^−/−^Tg mice.

### Dysregulated Treg phenotype and function in *Il2ra*^−/−^Tg mice

**Figure 3:**
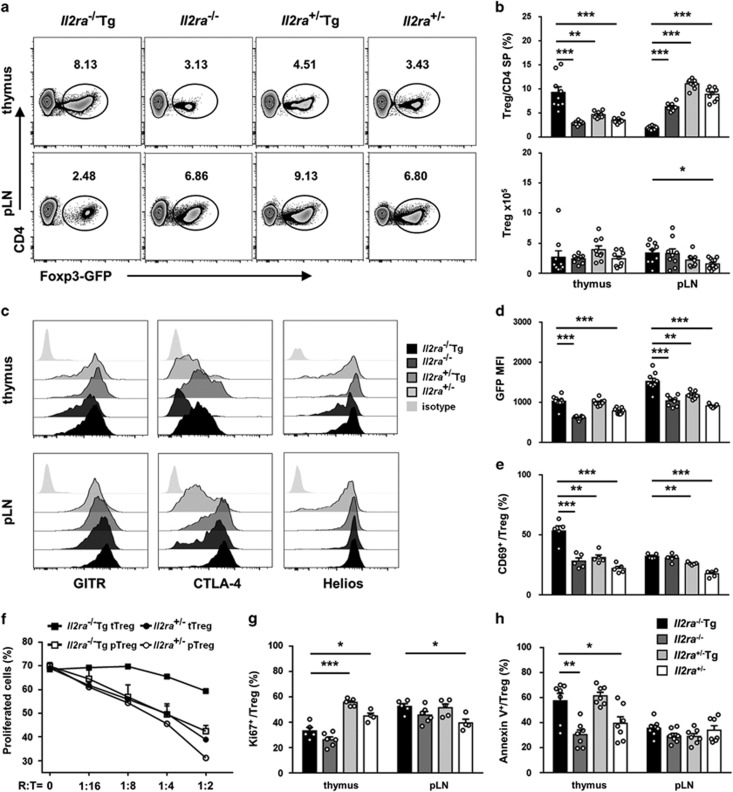
Dysregulated Treg percentage and function in *Il2ra*^−/−^Tg mice. (**a**) Representative flow cytometry result of Treg cell percentage in CD4 single-positive T cells in thymus and pLN of *Il2ra*^−/−^Tg and control mice. (**b**) Statistical analysis of Treg percentage and absolute number in thymus and pLN of *Il2ra*^−/−^Tg (*n*=9), *Il2ra*^−/−^ (*n*=9), *Il2ra*^+/−^Tg (*n*=9) and *Il2ra*^+/−^ (*n*=9) mice. (**c**) Expression of GITR, CTLA-4 and Helios of Treg cells in thymus and pLN from *Il2ra*^−/−^Tg and littermate control mice. (**d**) Foxp3 expression as indicated by GFP MFI of Treg cells in thymus and pLN of *Il2ra*^−/−^Tg (*n*=9), *Il2ra*^−/−^ (*n*=9), *Il2ra*^+/−^Tg (*n*=9) and *Il2ra*^+/−^ (*n*=9) mice. (**e**) Percentage of CD69^+^ Treg cells in thymus and pLN of *Il2ra*^−/−^Tg (*n*=5), *Il2ra*^−/−^ (*n*=5), *Il2ra*^+/−^Tg (*n*=5) and *Il2ra*^+/−^ (*n*=5) mice. (**f**) CFSE-labeled CD45.1^+^CD4^+^Foxp3-GFP^-^CD62L^+^ T cells were co-cultured with CD45.2^+^CD4^+^ Foxp3-GFP^+^ Treg cells from thymus and spleen/pLN of *Il2ra*^−/−^Tg and *Il2ra*^+/−^ mice in the presence of soluble anti-CD3 (2 *μ*g/ml) and anti-CD28 (1 *μ*g/ml) and mitomycin C treated supporter cells for 72 h. Percentage of proliferated cells among live target cells were analyzed. (**g**) Ki-67 staining of Treg cells in thymus and pLN of *Il2ra*^−/−^Tg (*n*=5), *Il2ra*^−/−^ (*n*=6), *Il2ra*^+/−^Tg (*n*=5) and *Il2ra*^+/−^ (*n*=4) mice. (**h**) Annexin V staining of Treg cells in thymus and pLN of *Il2ra*^−/−^Tg (*n*=7), *Il2ra*^−/−^ (*n*=7), *Il2ra*^+/−^Tg (*n*=7) and *Il2ra*^+/−^ (*n*=7) mice. Data are shown in mean±S.E.M. *P<0.05, **P<0.01, ***P<0.001. (Student's *t* test)

Treg percentage was increased in thymus but decreased in pLN in *Il2ra*^−/−^Tg mice at 3–4 weeks of age (Figure 3a). Increased thymus Treg cell percentage was likely due to thymus atrophy because it did not happen in 2 week *Il2ra*^−/−^Tg mice (Supplementary Figure 2a). Treg cell number did not change in the thymus but increased in pLN due to increase of cells in *Il2ra*^−/−^Tg mice (Figure 3b), which suggested that Treg generation from thymus was not affected in the absence of TGF-*β* and IL-2 signaling in T cells. GITR, CTLA-4 expression in peripheral rather than thymus Treg cells were elevated (Figure 3c), along with increased Foxp3 expression (Figure 3d). However, Treg cells from 1w *Il2ra*^−/−^Tg mice had decreased Foxp3 expression as well as decreased GITR, CTLA-4 expression, which was similar to *Il2ra*^−/−^ mice (Supplementary Figures 2d and e). Interestingly, Helios expression, a marker of activated Treg cells,^14^ was high in both thymus and peripheral Treg cells from *Il2ra*^−/−^Tg and control mice (Figure 3c). The percentage of CD69^+^ Treg cells was also increased in both thymus and peripheral Treg cells in *Il2ra*^−/−^Tg mice (Figure 3e). Suppressive function of *Il2ra*^−/−^Tg thymus Treg cells was decreased (Figure 3f). We suggested that decreased suppressive function may be due to decreased proliferation and increased apoptosis of *Il2ra*^−/−^Tg thymus Treg cells (Figures 3g and h). However, the proliferation of *Il2ra*^−/−^Tg peripheral Treg cells increased but they did not show increased apoptosis, and they maintained normal suppressive function *in vitro* compared with control mice (Figures 3f–h).

### Treg cells are skewed to Th1-like stable phenotype in *Il2ra*^−/−^Tg mice

**Figure 4:**
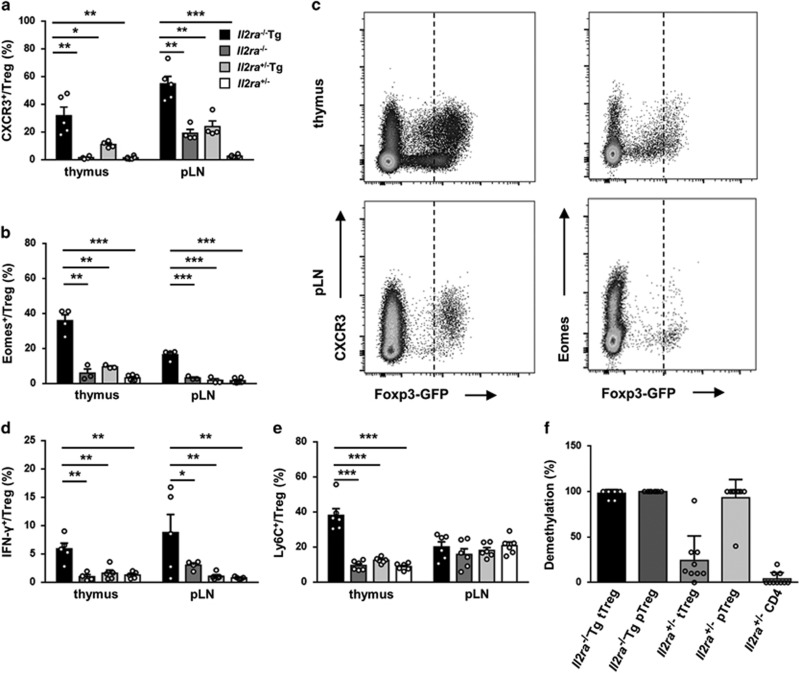
Treg cells are skewed to Th1-like stable phenotype in *Il2ra*^−/−^Tg mice. (**a**) Percentage of CXCR3^+^ Treg cells in thymus and pLN of *Il2ra*^−/−^Tg (*n*=5), *Il2ra*^−/−^ (*n*=4), *Il2ra*^+/−^Tg (*n*=4) and *Il2ra*^+/−^ (*n*=5) mice. (**b**) Percentage of Eomes^+^ Treg cells in thymus and pLN of *Il2ra*^−/−^Tg (*n*=4), *Il2ra*^−/−^ (*n*=3), *Il2ra*^+/−^Tg (*n*=3) and *Il2ra*^+/−^ (*n*=6) mice. (**c**) Representative flow cytometry result of Foxp3 and CXCR3, Eomes expression on CD4^+^CD8^−^ T cells from thymus and pLN of *Il2ra*^−/−^Tg and control mice. (**d**) IFN-*γ* secreting ability of thymus and pLN Treg cells from *Il2ra*^−/−^Tg (*n*=5), *Il2ra*^−/−^ (*n*=4), *Il2ra*^+/−^Tg (*n*=5) and *Il2ra*^+/−^ (*n*=5) mice. (**e**) Ly6C expression of thymus and pLN Treg cells from *Il2ra*^−/−^Tg (*n*=6), *Il2ra*^−/−^ (*n*=6), *Il2ra*^+/−^Tg (*n*=6) and *Il2ra*^+/−^ (*n*=6) mice. (**f**) Percentage of Foxp3 TSDR CpG demethylation of thymus and spleen/pLN Treg cells from *Il2ra*^−/−^Tg and *Il2ra*^+/−^ mice. Data are shown in mean±S.E.M. *P<0.05, **P<0.01, ***P<0.001. (Student's *t* test)

Treg cell phenotype can be regulated by cytokines in the microenvironment around them and further resulted in functional plasticity.^15^ As a consequence of increased serum levels of TNF-*α* and IFN-*γ* in *Il2ra*^−/−^Tg mice (Figure 1f), Th1 chemokine receptor CXCR3 (Figure 4a) and transcription factor Eomes (Figure 4b) expression were significantly increased in both thymus and peripheral *Il2ra*^−/−^Tg Treg cells. Interestingly, Treg cells expressing CXCR3 or Eomes had higher Foxp3 expression than CXCR3^−^ or Eomes^−^ Treg cells (Figure 4c). Besides, *Il2ra*^−/−^Tg Treg cells from thymus and pLN had a greater ability to secrete IFN-*γ* (Figure 4d). Ly6C expression on Treg cells negatively correlates with TCR signaling.^16^ We found Ly6C expression was increased in thymus but not in peripheral Treg cells in *Il2ra*^−/−^Tg mice, indicating aberrant TCR signaling during thymus Treg development (Figure 4e). Both thymus and peripheral Treg cells from *Il2ra*^−/−^Tg mice had increased Foxp3 TSDR CpG demethylation (Figure 4f), which was in accordance with their higher Foxp3 expression, suggesting a more stable phenotype.

### Defective Nrp-1^+^ Treg and Tfr development in *Il2ra*^−/−^Tg mice

**Figure 5:**
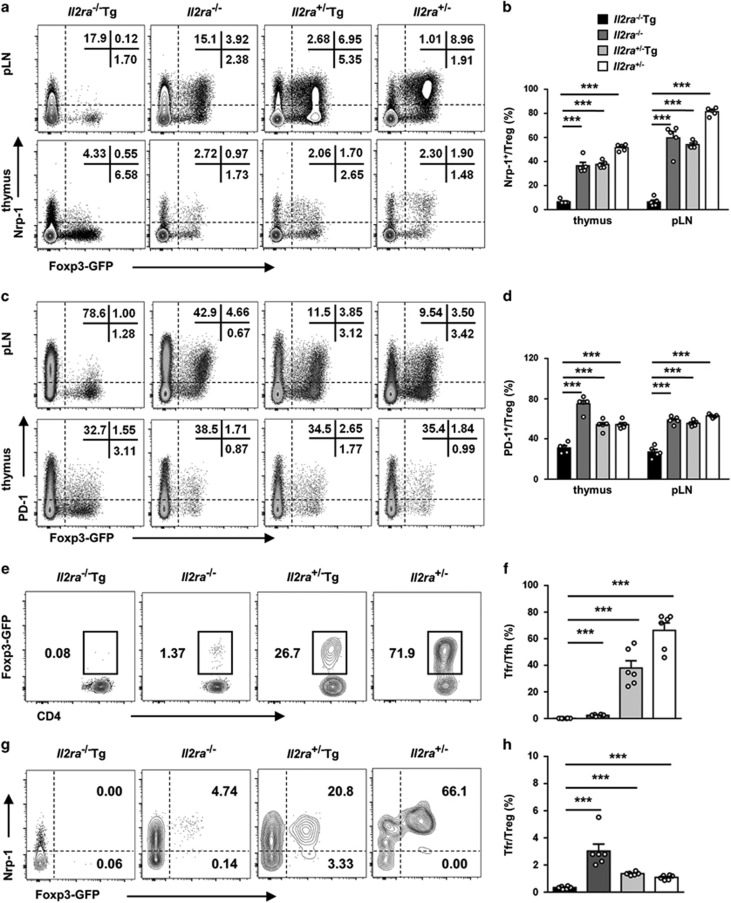
Defective Nrp-1^+^ Treg and follicular regulatory T-cell development in *Il2ra*^−/−^Tg mice. (**a**) Representative flow cytometry results of Nrp-1 expression on Treg and conventional CD4^+^ T cells from thymus and pLN of *Il2ra*^−/−^Tg and littermate control mice. (**b**) Nrp-1^+^ percentage of Treg in thymus and pLN from *Il2ra*^−/−^Tg (*n*=5), *Il2ra*^−/−^ (*n*=5), *Il2ra*^+/−^Tg (*n*=5) and *Il2ra*^+/−^ (*n*=5) mice. (**c**) Representative flow cytometry results of PD-1 expression on Treg and conventional CD4^+^ T cells from thymus and pLN of *Il2ra*^−/−^Tg and littermate control mice. (**d**) PD-1^+^ percentage of Treg in thymus and pLN from *Il2ra*^−/−^Tg (*n*=5), *Il2ra*^−/−^ (*n*=5), *Il2ra*^+/−^Tg (*n*=5) and *Il2ra*^+/−^ (*n*=5) mice. (**e**) Representative flow cytometry result of Tfr in Tfh cells in pLN of *Il2ra*^−/−^Tg and littermate control mice. Percentage of Tfr cells (**f**) in Tfh cells and (**h**) in Treg cells in pLN of *Il2ra*^−/−^Tg (*n*=6), *Il2ra*^−/−^ (*n*=6), *Il2ra*^+/−^Tg (*n*=6) and *Il2ra*^+/−^ (*n*=6) mice. (**g**) Nrp-1 and Foxp3 expression of Tfh cells in pLN of *Il2ra*^−/−^Tg and littermate control mice. Data are shown in mean±S.E.M. ***P<0.001. (Student's *t* test)

Based on the finding of abnormal Treg cell development in *Il2ra*^−/−^Tg mice, we raised the hypothesis that increased Tfh cell and enhanced germinal center reaction in *Il2ra*^−/−^Tg mice were due to a defective Tfr development. As Tfr cells develop from thymus Treg precursors,^17^ we first detected thymus Treg marker Nrp-1 expression on Treg cells. We found an almost complete loss of Nrp-1 expression on both thymus and peripheral Treg cells (Figures 5a and b). There was also a significant decrease of PD-1 expression on *Il2ra*^−/−^Tg Treg cells and an absence of the PD-1^hi^ population (Figures 5c and d). In addition, conventional CD4^+^ T cells from thymus or periphery expressed higher levels of Nrp-1 and PD-1 in *Il2ra*^−/−^Tg, *Il2ra*^−/−^ and *Il2ra*^+/−^Tg mice, indicating that Nrp-1 and PD-1 expression on Treg cells depended on both TGF-*β* and IL-2 signaling (Figures 5a and c).

We further found that the percentage of Tfr cells in Tfh cells was significantly decreased in *Il2ra*^−/−^Tg mice (Figures 5e and f). All Tfr cells were Nrp-1^hi^ in *Il2ra*^+/−^ mice, and most of them were Nrp-1^+^ in *Il2ra*^−/−^ and *Il2ra*^+/−^Tg mice. Nrp-1 expression on Tfr cells decreased when either IL-2 or TGF-*β* signaling was blocked (Figure 5g). Deficiency of Nrp-1 and PD-1 expression on Treg cells from *Il2ra*^−/−^Tg mice was intrinsic as Treg cells from 1w and 2w *Il2ra*^−/−^Tg mice also showed decreased Nrp-1 and PD-1 expression (Supplementary Figures 2b, c, f and g). Furthermore, the ability to develop into Tfr cells from *Il2ra*^−/−^Tg Treg cells was decreased compared with Treg cells from littermate control mice (Figures 5g and h). These data suggested that increased Tfh cell percentage and increased germinal center reaction may due to impaired Tfr development in*Il2ra*^−/−^Tg mice.

### T-cell activation and enhanced germinal center response in *Il2ra*^−/−^Tg mice are suppressed by wild-type Treg cells

**Figure 6:**
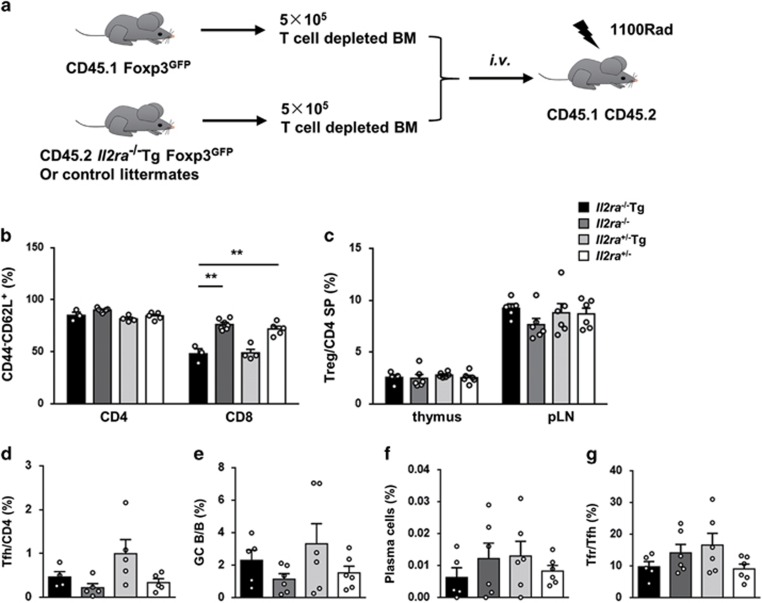
WT Treg cells suppress *Il2ra*^−/−^Tg T cells after bone marrow chimera. (**a**) Strategy of bone marrow chimera: 5 × 10^5^ T-cell depleted bone marrow cells from CD45.1 Foxp3^GFP^ mice were mixed with 5 × 10^5^ T-cell depleted bone marrow cells from CD45.2 *Il2ra*^−/−^Tg or littermate control mice and transferred intravenously to lethally irradiated CD45.1 CD45.2 recipient mice. Mice were killed 10 weeks after bone marrow transfer. (**b**) Naive T cell (CD44^−^CD62L^+^) percentage of pLN CD4^+^ and CD8^+^ T cells derived from *Il2ra*^−/−^Tg (*n*=3), *Il2ra*^−/−^ (*n*=4), *Il2ra*^+/−^Tg (*n*=4) and *Il2ra*^+/−^ (*n*=4) bone marrow. (**c**) Treg percentage in total CD4^+^ T cells after chimerism of *Il2ra*^−/−^Tg (*n*=5), *Il2ra*^−/−^ (*n*=6), *Il2ra*^+/−^Tg (*n*=6) and *Il2ra*^+/−^ (*n*=6) with WT bone marrow. (**d**) Tfh percentage of CD4^+^ T cells, (**e**) GC B percentage of B cells and (**f**) plasma cell percentage of total cells derived from *Il2ra*^−/−^Tg (*n*=5), *Il2ra*^−/−^ (*n*=6), *Il2ra*^+/−^Tg (*n*=6) and *Il2ra*^+/−^ (*n*=6) bone marrow. (**g**) Tfr cell percentage of Tfh cells after chimerism of *Il2ra*^−/−^Tg (*n*=4), *Il2ra*^−/−^ (*n*=5), *Il2ra*^+/−^Tg (*n*=5) and *Il2ra*^+/−^ (*n*=5) with WT bone marrow. Data are shown in mean±S.E.M. **P<0.01. (Student's *t* test)

As TGF-*β* and IL-2 signaling are blocked in all T cells, we used bone marrow chimera (Figure 6a) to confirm the dominant effect of TGF-*β* and IL-2 signaling deficiency on Treg cells. Mice reconstituted with *Il2ra*^−/−^Tg and CD45.1 wild-type (WT) bone marrow did not demonstrate tissue damage (data not shown). Naive cell percentage was high in both CD4^+^ and CD8^+^ T cells derived from *Il2ra*^−/−^Tg bone marrow and comparable with T cells derived from *Il2ra*^+/−^ bone marrow (BM), even though CD8^+^ T cells had a defective TGF-*β* signaling, demonstrating a decreased naive cell percentage (Figure 6b). There were almost no Treg cells derived from *Il2ra*^−/−^Tg and *Il2ra*^−/−^ bone marrow as they were less competitive due to IL-2 signaling deficiency, but total Treg cell percentage were comparable after bone marrow chimera (Figure 6c). These data indicated that *Il2ra*^−/−^Tg T-cell activation was suppressed by WT Treg cells.

Importantly, after chimeras with WT bone marrow, neither the Tfh cell percentage in CD4^+^ T cells nor GC B-cell percentage in B cells increased in *Il2ra*^−/−^Tg mice (Figures 6d and e). These data suggest that blocking TGF-*β* and IL-2 signaling in conventional T cells was not the main cause of increased Tfh cells and enhanced germinal center response in *Il2ra*^−/−^Tg mice. In addition, after chimerism with WT bone marrow, there were almost no plasma cells derived from *Il2ra*^−/−^Tg or control bone marrow (Figure 6f). Tfr percentage in Tfh cells were comparable after bone marrow chimera, suggesting that defective Tfr development was complemented by WT Treg cells, which suppressed the proliferation of Tfh cells derived from *Il2ra*^−/−^Tg bone marrow (Figure 6g). Thus, decreased Treg percentage and defective Tfr development due to TGF-*β* and IL-2 signaling blockade led to dysregulated germinal center responses and increased antibody secreting plasma cells and activated T cells, resulting in multi-organ inflammation (Supplementary Figure 3).

## Discussion

There is no issue more critical in autoimmunity than the dissection of mechanisms that are critical for immune tolerance.^10, 18, 19, 20, 21, 22, 23, 24, 25, 26^ Previous work have focused on the regulation of Treg cells, which are of crucial importance in preventing autoimmunity, by the cytokines TGF-*β* and IL-2. In this study, we thought to use *Il2ra*^−/−^Tg mice as a unique model to understand the synergetic role of TGF-*β* and IL-2 signaling on Treg cells. We found that *Il2ra*^−/−^Tg mice developed multi-organ inflammation, and *Il2ra*^−/−^Tg T cells were more pathogenic due to decreased Treg percentage but this could be rescued by WT Treg cells. More importantly, TGF-*β* and IL-2 signaling controlled the expression of PD-1 and Nrp-1 on Treg cells, as well as Treg development into Tfr cells.

We studied Treg cell development and function in a circumstance that they could still receive low levels of TGF-*β* and IL-2 signaling in *Il2ra*^−/−^Tg mice. In *Il2ra*^−/−^ mice, IL-2 can bind to its receptor *β* and *γ* to provide a low level of IL-2 signaling to Treg cells. Low IL-2 receptor signaling thresholds support the production of natural but not induced Treg cells.^27^ On the other hand, there is still weak TGF-*β* signaling in dnTGF*β*RII mice, and the abrogation of TGF-*β* signaling in T cells can be enhanced by increasing the copy number of dnTGF*β*RII gene.^28^ It is reported that TGF-*β* plays an important role in early Treg development because Lck-cre TGF*β*RI^f/f^ mice had negligible Treg cells before 1 week and increased Treg percentage thereafter due to increased IL-2 signaling.^7^ However, we found Treg phenotype of 1w *Il2ra*^−/−^Tg mice was similar to *Il2ra*^−/−^ mice, and 1w *Il2ra*^+/−^Tg mice had higher Treg percentage, indicating that IL-2 rather than TGF-*β* is important during neonatal Treg development. Besides, Treg cell percentage decreased in the periphery but increased in thymus compared with control mice at 3–4 weeks of age.

Treg cells can be activated by inflammatory cytokines.^15^ IFN-*γ* can induce Foxp3 expression and IFN-*γ* stimulated Treg cells express T-bet and CXCR3, which preferentially suppress type I inflammation.^29, 30^ IFN-*γ* or IL-27 can promote phosphorylation of STAT1 and T-bet, CXCR3, IFN-*γ* expression in Treg cells.^31^ Treg cells from thymus and spleen can be boosted by pathogenic T-cell-secreted TNF-*α* but not IL-2, which may be a critical mechanism for Treg cells in preventing autoimmune diseases.^32, 33, 34^ However, other studies reported that the function of TGF-*β* induced Treg cells can be impaired by TNF-*α* signaling.^35^ In our work, we also found an activated Treg phenotype in *Il2ra*^−/−^Tg mice. This was consistent with elevated serum concentrations of IFN-*γ* and TNF-*α*. In the absence of TGF-*β* signaling, Treg cells are more likely to be activated by TNF-*α* and maintain suppressive activity. However, thymic Treg cells from *Il2ra*^−/−^Tg mice were less activated, expressed low levels of Treg functional molecules and had less proliferation ability, resulting in decreased suppressive function *in vitro*.

Distinct from peripheral induced Treg cells, thymus-derived Treg cells (tTreg) have PD-1 expression.^36^ PD-1/PD-L1 interaction contributes to the generation of Treg cells from naive CD4^+^ T cells.^37^ PD-1 expressed on Treg cells can interact with PD-L1 expressed on T cells to mediate immune suppression.^24^ We found that CD4^+^ conventional T and CD8^+^ T cells from *Il2ra*^−/−^Tg mice expressed higher level of PD-1, while PD-1 expression on Treg cells was decreased. However, in bone marrow chimera experiments, PD-1 expression on conventional T cells was inhibited by WT Treg cells (data not shown). This indicated that PD-1 expression was differently regulated in conventional T cells and Treg cells. As both thymus and peripheral Treg cells from 1, 2 and 3 weeks of age *Il2ra*^−/−^Tg mice did not contain the PD-1^hi^ subset, high levels of PD-1 expression on Treg cells requires either TGF-*β* or IL-2 and were independent of inflammation.

During antigen recognition, Nrp-1 expression facilitates Treg interaction with DCs to suppress T-cell activation.^38^ On the other hand, semaphorin-4a ligation of Nrp-1 on Treg cells can maintain Treg stability and function through induction of PTEN.^39^ Although deficiency of Nrp-1 does not affect the ability to suppress autoimmunity and maintain immune homeostasis, deficiency of Nrp-1 on Treg cells leads to enhanced immune response in tumor model.^39, 40^ These suggest that Nrp-1 regulation of Treg cells needs further investigations. Nrp-1 expression on Treg cells is reported to distinguish tTreg and peripheral generated Treg cells, and is controlled by TGF-*β* signaling.^36, 41^ However, we found that block of either TGF-*β* or IL-2 could decrease Nrp-1 expression on Treg cells in 1, 2 and 3 weeks of age *Il2ra*^−/−^Tg mice, and Nrp-1 expression was lost when they were both blocked. These data suggest that Nrp-1 expression on Treg cells depends on both IL-2 and TGF-*β* signaling, and they act in a synergistic fashion.

Follicular regulatory T (Tfr) cells are a subset of Treg cells which share characteristics with Tfh cells in expressing Bcl-6 and CXCR5. Tfr develop from thymus Treg precursors and control humoral response by suppressing Tfh cell proliferation and germinal center B-cell development.^17, 42, 43^ Although the transcription factor Bcl-6 is critical for Tfr differentiation,^42^ little is known about mechanisms regulating their development from Treg precursors. In *Il2ra*^−/−^Tg mice, we supposed that there were no peripheral induced Treg cells in the absence of TGF-*β* signaling. We found that all Tfr cells were Nrp-1^+^ in *Il2ra*^+/−^ mice, but tTregs failed to express Nrp-1 in *Il2ra*^−/−^Tg mice. We speculate that TGF-*β* and IL-2 signaling may function through regulating Bcl-6 as well as Nrp-1 expression to control Tfr differentiation but the exact molecular mechanisms needs further studies. On the other hand, we found that although Tfh percentage were comparable in *Il2ra*^−/−^Tg and *Il2ra*^−/−^ mice, GC B-cell percentage and especially plasma cell percentage increased significantly. This suggests that controlling germinal center response by Tfr cells may suppress not only Tfh cell proliferation, but also their ability to induce antibody secreting plasma cells.

There is a balance between Th1 cells and Tfh cells.^44^ Similarly, we suppose if there is a balance between Th1-like Treg cells and Tfr cells. Eomes can compensate T-bet to induce IFN-*γ* production in Th1 cells, and expression of Eomes in CD4^+^ T cells restricts peripheral Foxp3 induction.^45, 46^ In our work, we reported upregulated expression of Eomes, CXCR3 on *Il2ra*^−/−^Tg Treg cells, which are associated with Th1-like Treg phenotype. In the meantime, *Il2ra*^−/−^Tg Treg cells had decreased PD-1, Nrp-1 expression and defective ability to differentiate into Tfr cells. This indicates that TGF-*β* and IL-2 are important in maintaining the balance between Th1-like Treg cells and Tfr cells, blocking TGF-*β* and IL-2 signaling will lead to preferentially differentiation of Th1-like Treg cells and defective development of Tfr cells. In summary, our data reveal a cytokine regulation of follicular regulatory T-cell development and function, and may provide a new therapeutic target for germinal center dependent autoantibody mediated autoimmune diseases.

## Material and Methods

### Mice

*Il2ra*^−/−^ mice (B6.129S4-Il2ratm1Dw), dnTGF-*β*RII mice (B6.Cg-Tg (Cd4-TGFBR2)16Flv/J) and CD45.1 congenic mice on a C57BL/6 J background were obtained from Jackson Laboratory (Bar Harbor, ME). Foxp3^GFP^ mice (Foxp3^tm2Ayr^) were kindly provided by Dr A.Y. Rudensky.^47^ dnTGF-*β*RII *Il2ra*^−/−^ (*Il2ra*^−/−^Tg) mice were obtained by crossing dnTGF-*β*RII mice with *Il2ra*^+/−^ mice. Littermates with genotypes of *Il2ra*^−/−^, dnTGF-*β*RII *Il2ra*^+/−^ (*Il2ra*^+/−^Tg) and *Il2ra*^+/−^ were used as control groups. Foxp3^GFP^ mice were crossed to them to enable the trace of Treg cells. *Il2ra*^−/−^Tg mice and control mice were studied at 1, 2 or 3–4 weeks of age. All mice were maintained under specific pathogen-free conditions at the Laboratory Animal Center, School of Life Sciences, University of Science and Technology of China (USTC). All animal experiments were approved by the USTC Animal Care and Use Committee.

### Histology

Liver, pancreas, lung and dorsal skin sections from *Il2ra*^−/−^Tg and control mice were immediately fixed in 4% paraformaldehyde after killing and embedded in paraffin. Tissue sections were then cut into 4*μ*m slices, deparaffinized and stained with hematoxylin and eosin for microscopic examination.

### Flow cytometry

Spleen, thymus, axillary, inguinal and brachial lymph nodes (pLN) were disrupted between two glass slides, suspended in PBS containing 0.2% BSA, and passed through a 74*μ*m nylon mesh. Red blood cells were depleted using Red blood lysis buffer. Cells were then counted manually on a hemocytometer. Fluorochrome-conjugated monoclonal antibodies were used including CD45.1 (A20), CD3 (17A2), CD4 (GK1.5), NK1.1 (PK136), CD19 (6D5), CD44 (IM7), CD62L (MEL-14), CD8*α* (19517), CD69 (H1.2F3), GITR (YGITR765), Ly6C (HK1.4), CXCR3 (CXCR3-173), CXCR5 (L138D7), PD-1 (29F.1A12), GL7 (GL7), CD138 (281-2), Neurophilin-1 (3E12), CTLA-4 (UC10-469), Helios (22F6), Ki-67 (16A8), IFN-*γ* (XMG1.2) (BioLegend, San Diego, CA, USA), or CD45.2 (104), B220 (RA3-6B2), CD8*α* (19517) (BD biosciences, San Diego, CA, USA) and Eomes (Dan11mag), CD95 (15A7) (eBioscience, San Diego, CA, USA). The protocol for surface molecule staining and intracellular cytokine measurement were performed as described.^48^ For Annexin V staining, cells were incubated with Allophycocyanin conjugated Annexin V (BioLegend) in Annexin V binding buffer (BioLegend) for 10 min after surface molecule staining, and then detected by flow cytometry. For transcription factors and CTLA-4 measurement, cells were first stained with antibodies against surface molecules, then fixed and permed using Foxp3 Staining Buffer Set (eBioscience), before antibodies against intracellular molecules were added. IgG isotype controls were used in parallel. Data were acquired using a FACSVerse flow cytometry (BD Immunocytometry Systems, San Jose, CA, USA) and analyzed with Flowjo software (Tree star, Inc., Ashland, OR, USA).

### Cell sorting and *in vitro* suppression assay

Regulatory T cells from either thymus (CD4^+^CD8^-^Foxp3-GFP^+^) or spleen and pLNs (CD4^+^Foxp3-GFP^+^) of *Il2ra*^−/−^Tg and control mice were sorted using BD FACS AriaI (BD Immunocytometry Systems) or MoFlo Astrios^EQ^ (Beckman Coulter, Brea, CA, USA). CD4^+^Foxp3-GFP^−^CD62L^+^ T cells were sorted from CD45.1 Foxp3^GFP^ mice and labeled with CFSE (2 *μ*M, Life Technologies, Waltham, MA, USA) for 10 min at 37 °C. CFSE-labeled T cells were suspended in T-cell culture medium (5 × 10^4^/well, RPMI 1640 (Gibco, Grand island, NY, USA) supplemented with 10% FBS (Millipore, Darmstadt, Germany), 2 mM l-glutamine (Gibco), 1 mM sodium pyruvate (Gibco), 25 mM HEPES free acid (Gibco), 55 *μ*M 2-mercaptoethanol (Gibco) and 100 U/ml Penicillin/Streptomycin (Hyclone, Logan, UT, USA)) and cultured with Treg cells at indicated ratio in the presence of soluble *α*-CD3 (2 *μ*g/ml, BioLegend), *α*-CD28 (1 *μ*g/ml, BioLegend) and Mitomycin C (Sigma, St Louis, MO, USA)-treated splenic supporter cells (1 × 10^5^/well) in a 96-well round bottom plate (Corning, NY, USA) for 3 days. Dead cells were excluded by DAPI staining and T-cell proliferation were assessed by CFSE dilution.

### Bone marrow chimeras

Recipient CD45.1/CD45.2 mice were lethally irradiated (11 Gy) and rested for 6 h before bone marrow transplantation.^11^ After depleting T cells by cell sorting using MoFlo Astrios^EQ^, BM cells from CD45.1 Foxp3^GFP^ mice were mixed 1:1 (5 × 10^5^: 5 × 10^5^) with cells from *Il2ra*^−/−^Tg Foxp3^GFP^ or control mice and transferred intravenously into recipient mice. Mice were killed 10 weeks after bone marrow transplantation.

### DNA methylation analysis

Treg cells (CD8^−^CD4^+^Foxp3-GFP^+^) from thymus and spleen/pLN of *Il2ra*^−/−^Tg and *Il2ra*^+/−^mice were sorted. Genomic DNA from 5 × 10^4^ Treg cells were extracted and bisulfite converted using EZ DNA Methylation-Direct Kit (Zymo Research, CA, USA). PCR amplification were performed using TaKaRa EpiTaq^TM^ HS (for bisulfite treated DNA) (TaKaRa Bio Inc., Shiga, Japan). Primers for *Foxp3* TSDR were forward: 5′-TTTTGGGTTTTTTTGGTATTTAAGA-3′ reverse: 5′-TTAACCAAATTTTTCTACCATTAAC-3′. The amplification conditions were 95 °C for 15 s and 40 cycles of 95 °C for 25 s, 55 °C for 30 s, 72 °C for 30 s and a final extension step of 7 min at 72 °C. PCR products were separated using agarose gel, cloned into pGEM-T Vector (Promega, WI, USA) and transfected competent DH5*α*. 8-12 clones were picked and plasmid DNA were sequenced.

### Enzyme-linked immunosorbent assay

Serum concentration of IgG1, IgG2a, IgG2b, IgG3 and IgM were detected by Ready-Set-Go! ELISA kits (eBioscience).

### Cytometric bead array

Serum concentrations of IFN-*γ*, TNF-*α*, IL-4 and IL-17A from *Il2ra*^−/−^Tg and control mice were measured with a cytometric bead array kit (Mouse Th1/Th2/Th17 CBA kit, BD Biosciences), using a FACSVerse flow cytometer with CBA software (BD Biosciences).

### Statistical analysis

Statistical significance was analyzed using a two-tailed unpaired Student's *t* test in GraphPad Prism 5 (GraphPad Software, San Diego, CA, USA). All results were from at least two independent experiments and error bars represented mean±s.e.m.

## Abbreviations

Bcl-6: B-cell lymphoma 6
BM: bone marrow
CTLA-4: cytotoxic T-lymphocyte-associated protein 4
CXCR5: C-X-C chemokine receptor type 5
Eomes: Eomesodermin
Foxp3: forkhead box P3
GC: germinal center
GITR: glucocorticoid-induced TNFR family related gene
Nrp-1: Neuropilin-1
PD-1: programmed cell death protein 1
pLN: peripheral lymph nodes
Tfh cells: follicular helper T cells
Tfr cells: follicular regulatory T cells
TNF-*α*: tumor necrosis factor alpha
Treg cells: regulatory T cells
TSDR: Treg-specific demethylated region
tTreg: thymus-derived Treg cells
WT: wild type
