# Supplementary material for: Block of both TGF-β and IL-2 signaling impedes Neurophilin-1+ regulatory T cell and follicular regulatory T cell development
Source: Cell Death Dis. 2016 Oct 27;7(10):e2439–. doi: 10.1038/cddis.2016.348 (PMC5134002; doi:10.1038/cddis.2016.348)
Supplement: Supplementary Information [file cddis2016348x1.docx]

**SUPPLEMENTARY INFORMATION**

**Block of both TGF-β and IL-2 signaling impedes Neuropilin-1^+^ regulatory T cell and follicular regulatory T cell development**

Liang Li^1,*^, Shu-Han Yang^1,*^, Yuan Yao^1^, Yu-Qing Xie^1^, Yan-Qing Yang^1^, Yin-Hu Wang^1^, Xue-Ying Yin^1^, Hong-Di Ma^1,#^, M. Eric Gershwin^2^ and Zhe-Xiong Lian^1,3,#^

^1^Liver Immunology Laboratory, Institute of Immunology and School of Life Sciences, University of Science and Technology of China, Hefei 230027, China; ^2^Division of Rheumatology, Allergy and Clinical Immunology, University of California at Davis School of Medicine, Davis, CA 95616, USA; ^3^Innovation Center for Cell Signaling Network, Hefei National Laboratory for Physical Sciences at Microscale, Hefei 230027, China.

^*^ Both authors contributed equally to the manuscript.

**Supplementary Figure 1**. Phenotype of 3-4 week *Il2ra*^-/-^Tg and control mice. (**a**) Photograph of 3-4w *Il2ra*^-/-^Tg and littermate control mice. Photograph of peripheral lymph nodes (**b**), spleen (**c**) of *Il2ra*^-/-^Tg and littermate control mice. (**d**) Representative H&E staining (100×) of liver, pancreas, lung and dorsal skin from 3-4w *Il2ra*^-/-^Tg and littermate control mice.

**Supplementary Figure 2**. Treg phenotype of 1 week and 2 week *Il2ra*^-/-^Tg and control mice. (**a**) Treg percentage of CD4 SP T cells in thymus and pLN and of 2 week *Il2ra*^-/-^Tg (n=6), *Il2ra*^-/-^ (n=5), *Il2ra*^+/-^Tg (n=6) and *Il2ra*^+/-^ (n=6) mice. (**b**) Nrp-1^+^ percentage of Treg in pLN and thymus from 2 week *Il2ra*^-/-^Tg (n=3), *Il2ra*^-/-^ (n=4), *Il2ra*^+/-^Tg (n=5) and *Il2ra*^+/-^ (n=4) mice. (**c**) PD-1^+^ percentage of Treg in thymus and pLN from 2 week *Il2ra*^-/-^Tg (n=3), *Il2ra*^-/-^ (n=4), *Il2ra*^+/-^Tg (n=3) and *Il2ra*^+/-^ (n=4) mice. Representative flow cytometry result of (**d**) GITR and (**e**) CTLA-4 expression on Treg cells from thymus and pLN of 1 week *Il2ra*^-/-^Tg and control mice. **(f) Nrp-1^+^ and (g) PD-1^+^ percentage of Treg in pLN and thymus from 1 week *Il2ra*^-/-^Tg (n=4), *Il2ra*^-/-^ (n=3), *Il2ra*^+/-^Tg (n=5) and *Il2ra*^+/-^ (n=6) mice.** Data are shown in mean ± S.E.M. **P* < 0.05, ***P* < 0.01, ****P* < 0.001. (Student's *t*-test)

**Supplementary Figure 3**. Block of both TGF-β and IL-2 signaling impedes Nrp-1^+^ regulatory T cell and follicular regulatory T cell development. When TGF-β and IL-2 signaling were blocked, Treg cells decreased their expression of PD-1 and Nrp-1 expression during development in the thymus. They also had increased CXCR3 and Eomes expression and increased TSDR demethylation. They failed to contact with DC and effector T cells to prevent excessive activation of effector T cells s in early age of mice, and T cells produced IFN-γ and TNF-α to activate Treg cells and enhance their suppressive ability in the periphery. On the other hand, they failed to differentiate into follicular regulatory T cells to suppress germinal center response when TGF-β and IL-2 signaling were blocked. This resulted in Tfh cell and GC B cell proliferation, and B cell differentiation into plasma cells to produce autoantibodies.
