# Supplementary figures and images for: Block of both TGF-β and IL-2 signaling impedes Neurophilin-1+ regulatory T cell and follicular regulatory T cell development
Source: Cell Death Dis. 2016 Oct 27;7(10):e2439–. doi: 10.1038/cddis.2016.348 (PMC5134002; doi:10.1038/cddis.2016.348)

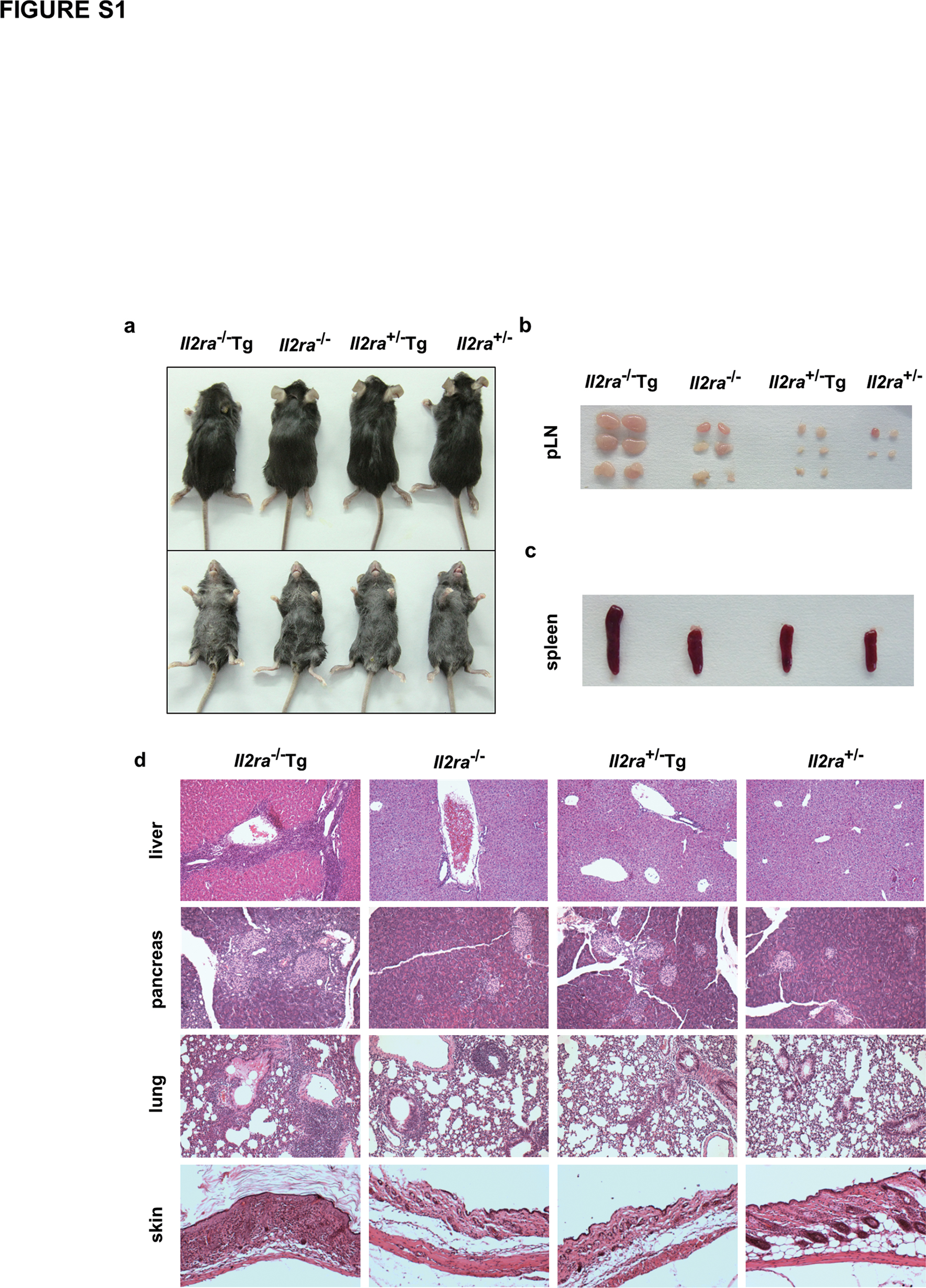

Supplement: Supplementary Figure 1 [file cddis2016348x2.tif]

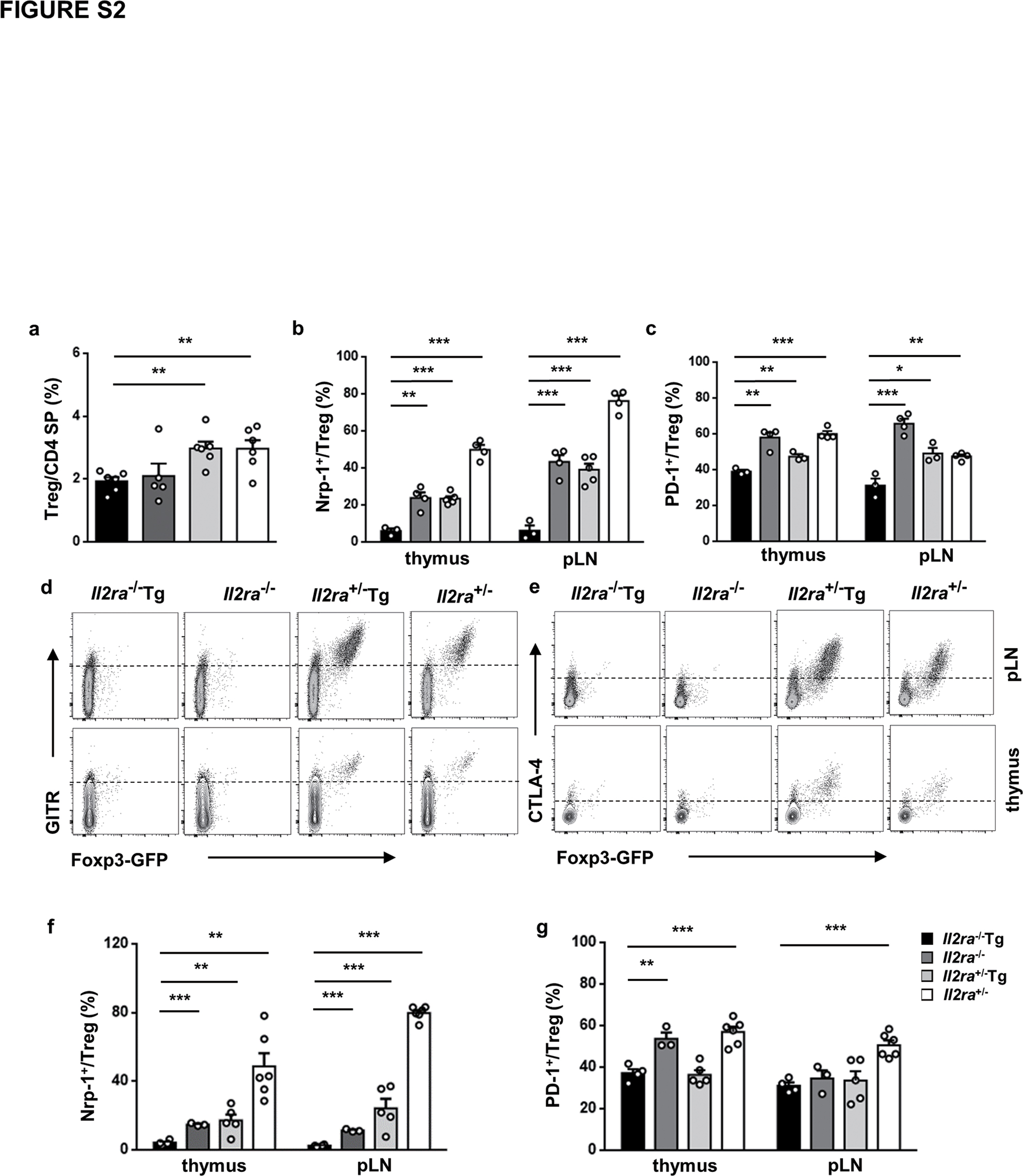

Supplement: Supplementary Figure 2 [file cddis2016348x3.tif]

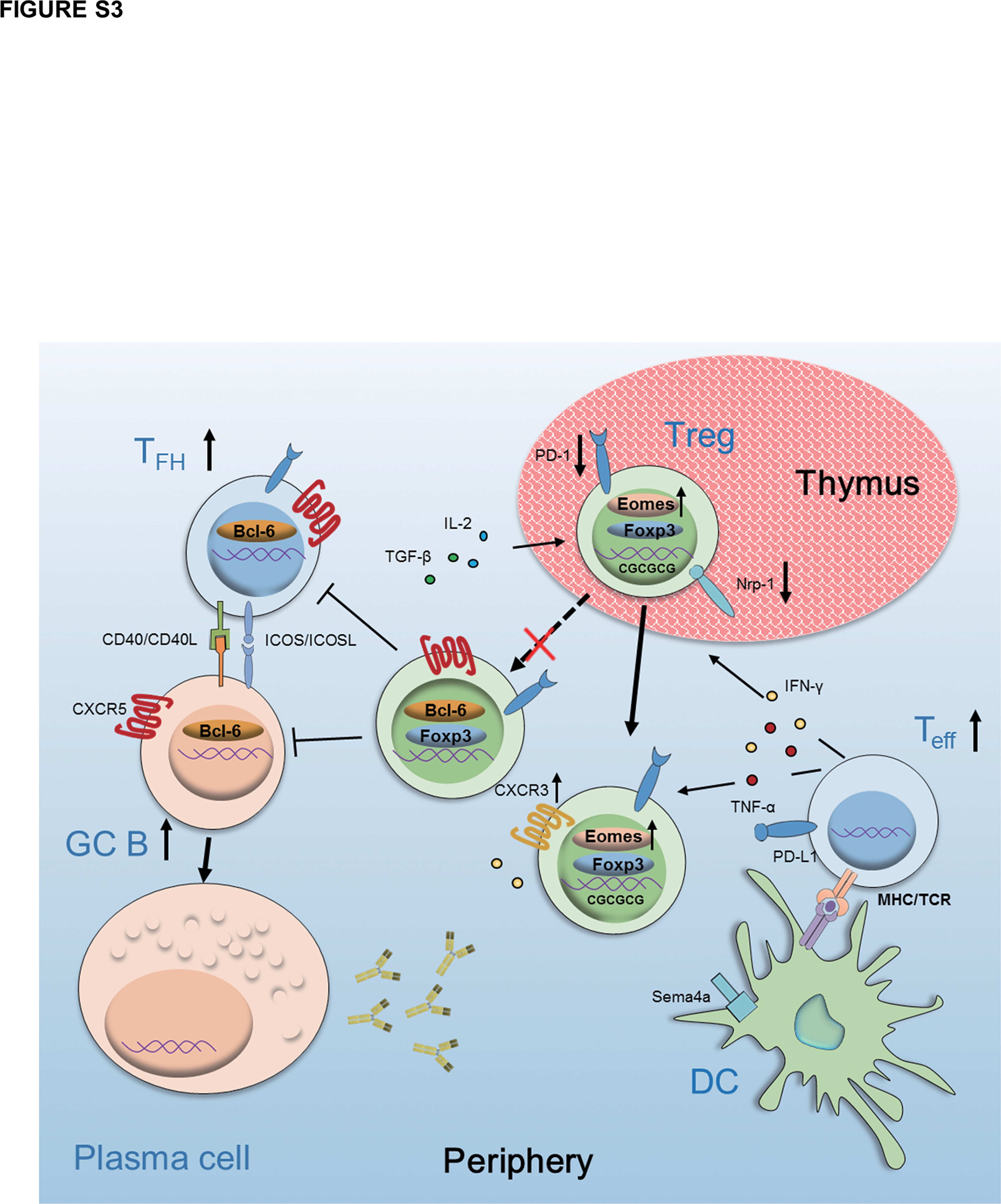

Supplement: Supplementary Figure 3 [file cddis2016348x4.tif]
